# The histone demethylase LSD1 regulates inner ear progenitor differentiation through interactions with Pax2 and the NuRD repressor complex

**DOI:** 10.1371/journal.pone.0191689

**Published:** 2018-01-25

**Authors:** Dharmeshkumar Patel, Atsushi Shimomura, Sreeparna Majumdar, Matthew C. Holley, Eri Hashino

**Affiliations:** 1 Department of Otolaryngology-Head and Neck Surgery and Stark Neurosciences Research Institute, Indiana University School of Medicine, Indianapolis, Indiana, United States of America; 2 Department of Biomedical Science, University of Sheffield, Sheffield, United Kingdom; Harvard University, UNITED STATES

## Abstract

The histone demethylase LSD1 plays a pivotal role in cellular differentiation, particularly in silencing lineage-specific genes. However, little is known about how LSD1 regulates neurosensory differentiation in the inner ear. Here we show that LSD1 interacts directly with the transcription factor Pax2 to form the NuRD co-repressor complex at the Pax2 target gene loci in a mouse otic neuronal progenitor cell line (VOT-N33). VOT-N33 cells expressing a Pax2-response element reporter were GFP-negative when untreated, but became GFP positive after forced differentiation or treatment with a potent LSD inhibitor. Pharmacological inhibition of LSD1 activity resulted in the enrichment of mono- and di-methylation of H3K4, upregulation of sensory neuronal genes and an increase in the number of sensory neurons in mouse inner ear organoids. Together, these results identify the LSD1/NuRD complex as a previously unrecognized modulator for Pax2-mediated neuronal differentiation in the inner ear.

## Introduction

Mechanosensitive hair cells and afferent spiral ganglion neurons in the inner ear are derived from multipotent otic progenitors during embryonic development. Elucidating the transcriptional network associated with differentiation of these cell types has been an area of intense investigation over the past decade [1, 2]. While a number of transcription factors essential for normal development of the inner ear have been identified, little is known about how these transcription factors epigenetically control inner ear development [3–8].

Epigenetic modifications have emerged as a key mechanism controlling the processes of cellular differentiation and cell fate specification. One such modification is the methylation of lysine on the tails of the core histones H3 and H4 [9]. Methylation at these sites has been associated with transcriptional activation or repression [10, 11], and the biological effects of methylation depend strongly on the lysine residues. In most cases, methylation of histone H3 at lysine 4 is associated with enhancers/promoters and linked to transcriptional activation, whereas methylation of histone H3 at lysine 9 is a hallmark of transcriptionally inactive heterochromatin [10–12]. In addition, the level of lysine methylation, such as mono-, di-, or tri-methylation, correlates with either transcriptional activation or repression. For instance, mono-methylation of histone H4 at lysine 20 is associated with transcriptional activation [13]. In contrast, tri-methylation of histone H4 at lysine 20 on promoters is associated with transcriptional repression [13, 14].

Histone lysine methylation has been shown to be dynamically regulated by both methyltransferases and demethylases. Lysine-specific demethylase 1 (LSD1) is the first discovered histone demethylase [15]. This enzyme is associated with different protein complexes and functions as a transcriptional activator or repressor in a target-specific manner. For example, LSD1 promotes transcriptional activation via interactions with androgen nuclear receptor (AR). As part of this complex, LSD1 removes repressive histone marks on lysine 9 of histone H3 (H3K9), thereby leading to derepression of its target genes [16, 17]. LSD1 also participates in gene repression as part of the corepressor for element-1-silencing transcription factor (CoREST), nucleosome remodeling and histone deacetylase (NuRD), or silent mating type information regulation 1 (SIRT1) repressor complexes [18–20]. Consistent with its role in transcription repression, LSD1 demethylates histone H3 at lysine 4 (H3K4), which is strongly associated with active transcription [15]. The NuRD cofactors including LSD1 are present throughout the organ of Corti from E18.5 until P4 [21]. These cofactors are completely absent by P7, detectable again at P8, and continue to be present through P21. The temporal expression pattern of the NuRD complex suggests that histone methylation might be involved in mechanisms controlling the developmental processes of the inner ear.

The paired homeodomain transcription factor Pax2 plays a critical role in the normal development of the inner ear [22]. During inner ear development, Pax2 is expressed in the ventral region of the otic vesicle, from which the cochlea, vestibular organ, and sensory neurons arise [23]. Two mutant alleles of *Pax2*, characterized by a knockout and a frameshift mutation (*Pax2*^*1Neu*^), result in agenesis or severe malformation of the cochlea and spiral ganglion, respectively [24]. *Pax2*-knockout mice exhibit severe cochlear defects as well as absence of the spiral ganglion [25]. The *Pax2*^*1Neu*^ mutation is identical to a mutation in a human family with renal coloboma syndrome, in which the spiral ganglion is not developed. In addition, the ventral cochlear chamber is enlarged with a distinct saccule and occasional absence of the utricle and ampullae [26].

In the present study, we identified LSD1/NuRD complex as a novel interaction partner of Pax2 and analyzed the function of the LSD1/NuRD complex in differentiation of otic progenitor cells toward sensory cell types. Our data suggest that epigenetic gene inactivation induced by the LSD1/NuRD complex is essential for the maintenance of multipotent otic progenitors prior to cell fate specification.

## Materials and methods

### Cell culture, transfection and stimulation

The mouse ventral otocyst-derived cell line (VOT-N33 –hereafter N33) [27, 28] was cultured under proliferating conditions in MEM (with Earle’s salt, Sigma) supplemented with 10% fetal bovine serum, 100 U/ml penicillin-streptomycin, 1% Glutamax (Gibco) and 50 U/ml mouse recombinant gamma interferon (Peprotech) in 5% CO_2_/95% air at 33°C. To induce differentiation, N33 cells were cultured in equal mixture of Neurobasal (Gibco) and Advance DMEM/F12 medium (Gibco) supplemented with 100 U/ml penicillin-streptomycin, 1% Glutamax (Gibco), 1% N-2 Supplement (Gibco) and 2% B27 Supplement (Gibco) in 5% CO_2_/95% air at 39°C. To make stable cells, the PRS4-enhanced green fluorescence protein (EGFP) construct containing the neo gene (kindly provided by Gregory Dressler, University of Michigan), described in [29], was linearized using the ApaL1 restriction enzyme (NEB). The linearized PRS4-EGFP construct was transfected into N33 cells using Lipofectamine LTX (Invitrogen) and stably transfected cell clones were selected using Geneticin (G418, Invitrogen) at 500 μg/ml. Where indicated, cells were stimulated with 400 ng/ml Trichostatin A (TSA, Millipore), 0.25 mM Tranylcypromine (TCP, Tocris), and 10 μM LSD1-C12 (Xcess Bioscience Inc).

### Antibodies

The following primary antibodies were used for western blotting: anti-PAX2 (#H00005076-M01, Abnova and #71–6000, Invitrogen), anti-LSD1 (#17721, Abcam), anti-MTA1 (#71153, Abcam), anti-Mi-2/CHD4 (#72418, Abcam), anti-HDAC-1 (#06–721, EMD Millipore), anti-HDAC-2 (#04–229, EMD Millipore), anti-MBD3 (#157464, Abcam), anti-RBBP4 (#79416, Abcam), anti-CtBP1 (#612042, BD Biosciences), anti-CtBP2 (#612044, BD Biosciences), anti-CoREST (#07–455, EMD Millipore), anti-AR (#133273, Abcam), anti-GFP (#290, Abcam), anti-β-actin (#A5441, Sigma), anti-NeuroD1 (#16507, Abcam), anti-acetyl histone H3 (#06–599, Millipore), and anti-histone H3 (#1791, Abcam).

The following primary antibodies were used for immunofluorescent staining: anti-PAX2 (#71–6000, Invitrogen), anti-LSD1 (#17721, Abcam), anti-Tuj1 (Covance), anti-PTIP ((#A300-370A, Bethyl laboratories), anti-MLL3 (#32581, Abcam), and anti-NeuroD1 (#16507, Abcam).

The following primary antibodies were used for chromatin immunoprecipitation (ChIP): anti-Pax2 (#71–6000, Invitrogen), anti-LSD1 (#17721, Abcam), anti-MBD2/3 (#28743, SantaCruz Biotechnology), anti-CHD4 (#72418, Abcam), anti-HDAC1 (#06–721, EMD Millipore), anti-HDAC2 (#04–229, EMD Millipore), anti-MTA1 (#71153, Abcam), anti-RBBP4 (#79416, Abcam), anti-CoREST (#07–455, EMD Millipore), anti-CtBP1 (#612042, BD Biosciences), anti-CtBP2 (#612044, BD Biosciences), anti-MLL3 (#32581, Abcam), anti-GRG4 (#64833, Abcam), anti-mono-methyl histone H3 (#8895, Abcam), anti-di-methyl histone H3 (#7766, Abcam), and anti-tri-methyl histone H3 (#8580,Abcam).

### FACS

At day 14 after transfection, exponentially growing N33 cells were harvested using 0.25% trypsin/EDTA and resuspended in cold phosphate buffer saline (PBS) at 1 X 10^7^ cells/ml. The collected cells were then sorted on an iCyt & Sony-Reflection cell sorter (Sony Biotechnology). EGFP-positive cells were collected using a 530/40 bandpass filter. Auto fluorescence of untransfected cells was detected as a control. Data were analyzed using FlowJo version 10.0.6 software (FlowJo, LLC). Post-sorting, the cells were collected in the maintenance medium with gamma interferon and cultured at 33°C under the selective pressure of G418 for approximately two months.

### Co-immunoprecipitation and western blotting

To prepare nuclear proteins for immunoprecipitation, N33 cells were resuspended in Buffer A (10 mM 4-(2-hydroxyethyl)-1-piperazineethanesulfonic acid (HEPES), pH 7.9, 10 mM KCl, 0.1 mM EDTA, 1 mM DTT, protease inhibitor mixture, and phosphatase inhibitor mixture), kept on ice for 15 min, and subsequently extracted in 0.5% Nonidet P-40. The extracts were homogenized with the Dounce homogenizer with 10 up-down strokes and centrifuged at 500 x g for 5 min at 4°C. The pellets were resuspended in buffer B (20 mM HEPES, 1.5 mM MgCl2, 420 mM NaCl, 0.2 mM EDTA, 25% glycerol, 1 mM DTT, protease inhibitor, and phosphatase inhibitors), incubated for 1 h with shaking to extract nuclear protein, and then centrifuged for 15 min at 18,000 × *g*. The supernatants were used as the nuclear extract.

For immunoprecipitation, the cell lysate was pre-cleared with protein G sepharose bead (Sigma-Aldrich). The precleared supernatants were incubated with either anti-Pax2 (#71–6000, Invitrogen) or anti-LSD1 (#17721, Abcam) antibodies. On the following day, protein G Sepharose bead (Amersham Biosciences) were added and incubated at 4°C for 2 h. The precipitates were washed three times with 10 mM Tris-HCl, pH 7.5, 75 mM NaCl, 1 mM EDTA, 6% glycerol, 1% NP-40, 1 mM DTT, protease inhibitor mixture, and phosphatase inhibitor mixture.

For extraction of the total protein from N33 cells, the cells were lysed in RIPA buffer (10 mM Tris-HCl, pH 7.4, 150 mM NaCl, 1 mM EDTA, 1% Nonidet P-40, 0.1% sodium deoxycholate, 0.1% sodium dodecyl sulfate (SDS), protease inhibitor mixture, and phosphatase inhibitor mixture). The extracts were subjected to centrifugation at 18000 x g at 4°C for 10 min in a microcentrifuge to remove insoluble debris and chromosomal DNA.

The extracted proteins and immunoprecipitates were separated by SDS-polyacrylamide gel electrophoresis (SDS-PAGE) and subjected to a western blotting assay with primary antibodies. Antibody binding was detected using horseradish peroxidase-conjugated secondary antibody (SantaCruz Biotechnology) and a Super Signal West Femto Chemiluminescence system (Thermo Scientific).

### Immunofluorescent staining

Animal experiments were approved by the Indiana University Institutional Animal Care and Use Committee, and conducted in accordance with the Regulations for the Management of Laboratory Animals at Indiana University. The mouse embryos at embryonic day (E) 9.5–11.5 were fixed with 4% paraformaldehyde (PFA) in 0.1 M phosphate buffer. The fixed embryos were cryoprotected in graded (20–40%) sucrose concentrations, embedded in OCT compound, frozen in liquid nitrogen, and then sectioned into 10-μm-thick cryosections. N33 cells were cultured on cover glass or in chambered slides and then fixed with 4% PFA in PBS for 20 min.

For immunofluorescent staining, frozen sections and fixed cells were incubated with blocking buffer (10% goat or horse serum and 0.1% Triton X-100 in PBS) for 1 h to block non-specific binding. Then, sections and cells were incubated with primary antibodies in blocking buffer for 1 h. After washing with PBS, the sections and cells were incubated for 45 min with secondary antibodies labeled with Alexa Fluor 488 or 568. DAPI counterstain was used to visualize cellular nuclei. The immunostained sections were examined under a confocal laser scanning system (Olympus FV1000 MPE).

### RNA isolation and quantitative RT-PCR

Extraction of total RNA and quantitative RT-PCR analyses were performed as described previously [30, 31]. To remove DNA contamination from RNA samples, an on-column DNase digestion step was performed according to the manufacturer's instructions (Qiagen). Complementary DNA (cDNA) was synthesized using Omniscript reverse transcriptase (Qiagen) and oligo-dT primers. Quantitative RT-PCR was done in the ABI PRISM 7900HT Sequence Detection System (Applied Biosystems) using the SYBR Green PCR Master Mix kit (Applied Biosystems). In brief, a master mixture was prepared, containing cDNA, SYBR Green PCR Master Mix, primers (Table 1), and uracil N-glycosylase. The amplification conditions for 45 cycles consisted of denaturation at 94°C for 15 s, annealing at 60°C for 30 s, and extension at 60°C for 30 s. The expression levels were normalized to *L27* mRNA expression.

**Table 1:** PCR primers used for gene expression analyses.

Primer	Sequence
L27-F	CGTCATCGTGAAGAACATTG
L27-R	CATGGCAGCTGTCACTTTC
Polr2a-F	CGGCAGTACGCAAATTCAC
Polr2a-R	TGGACAGAAACATTAAGAGGTTCA
Ngn1-F	TCGGCTTCAGAAGACTTCAC
Ngn1-R	GTGGTATGGGATGAAACAGG
NeuroD-F	CCTGATCTGGTCTCCTTCGT
NeuroD-R	AAGAAAGTCCGAGGGTTGAG
Phox2b-F	CCGCAGTTCCATACAAACTC
Phox2b-R	CCCTCTCCAACTCTTTGAGC
Tlx3-F	CAGAAGAGCCTCAACGATTC
Tlx3-R	GACCTTGGAACTGTCCTCCT
Brn3a-F	GGATGAAATTCTCTGCCACTT
Brn3a-R	CAACAACTTCACGGTTCTCC
Pax2-F	GAGAAGGGGTCTGCTTTGC
Pax2-R	CTGCCTATGACCGCCACTA
c-Myc-F	TAGTGCTGCATGAGGAGACA
c-Myc-R	GTTTGCCTCTTCTCCACAGA

### Chromatin immunoprecipitation (ChIP)

Cells were fixed for 10 min with 1% formaldehyde (Electron Microscopy Science # 15710-S) in culture medium. Cross-linking was stopped with 125 mM glycine. The cross-linked cells were harvested by centrifugation. The cell pellet was resuspended in 0.3 ml cell lysis buffer (50mM TrisHCl pH 8.0, 10mM EDTA, 1% SDS and 1X protease inhibitor cocktails (Roche #11836170001), and sonicated 6 x 15 s pulses using a Bioruptor sonication device from Diagenode at 20% power to achieve an average DNA fragment size of 500 bp. The sheared chromatin was centrifuged at 20000 X g for 10 minutes at 4°C. Supernatant was diluted with dilution buffer (1% Triton X-100, 2 mM EDTA, 150 mM NaCl and 20 mM Tris-HCl, pH 8,0), and then immunoprecipitated with normal IgG for negative controls or with relevant primary antibody. After overnight incubation at 4°C, the samples were incubate with protein G magnetic beads (Active motif # 53033) for 4 h at 4°C with gentle shaking. Magnetic bead-antibody-chromatin complexes were subsequently washed once in TSE I buffer (0.1% SDS, 1% Triton X-100, 2 mM EDTA, 20 mM Tris-HCl, pH 8.0, 150 mM NaCl), once in TSE II buffer (0.1% SDS, 1% Triton X-100, 2 mM EDTA, 20 mM Tris-HCl, pH 8.0, 500 mM NaCl), once in TSE III buffer 0.25 M LiCl, 1% NP-40, 1% deoxycholate, 1 mM EDTA, 10 mM Tris-HCl, pH 8.0), and finally three times in Tris-EDTA. To elute the antibody-bound chromatin, the beads were incubated in Elution buffer 1% SDS, 0.1 M NaHCO_3_) at 25°C for 1 h with shaking. The protein-DNA cross-link was reversed by heating at 65°C overnight. The shared chromatin was purified using QIAquick gel extraction kit (Qiagen). For ChIP samples, quantitative PCR was performed using the SYBR Green PCR Master Mix kit (Applied Biosystems) and primers (Table 2) as described above.

**Table 2:** PCR primers used for ChIP assays.

Primer	Sequence
PRS4-F	GCTACCGGACTCAGATCTCG
PRS4-F	TGCGAAGTGGACCTCGGACC
GAPDH-F	TGAGAGAGGCCCAGCTACTC
GAPDH-R	TTATAGGAACCCGGATGGTG

### Organoid culture

Inner ear organoids were generated from mouse embryonic stem cells according to previously described methods [31, 32]. At day 8 after the start of differentiation, embryonic stem cell-derived organoids were treated with 10 μM LSD1-C12 or DMSO as a negative control. After 48 h exposure to LSD1-C12 or DMSO, the organoid samples were fixed and processed for immunofluorescence as described above.

### Statistical analysis

The data presented are means of three independent experiments. Statistical evaluation was performed with Student’s *t*-test. *P*-values smaller than 0.05 were considered statistically significant and indicated on graphs with asterisks.

## Results

### Pax2 and LSD1 are co-expressed in otic progenitors *in vivo* and *in vitro*

**Fig 1:**
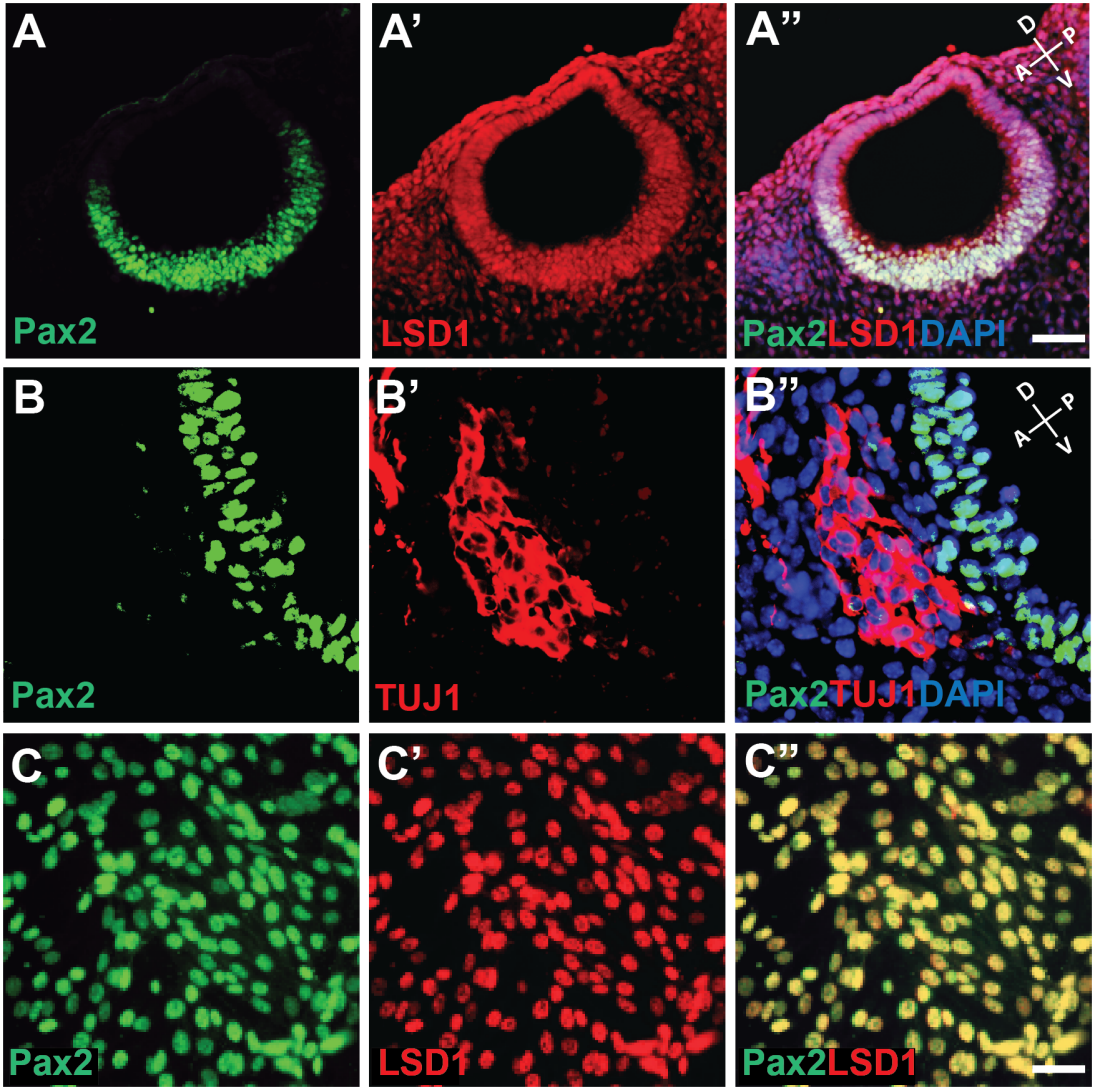
*In vivo* and *in vitro* expression of Pax2 and LSD1 in otic progenitors. (A-A”) Mouse E10.5 otic vesicle stained with Pax2 (green) and LSD1 (red) antibodies. (B-B”) Mouse embryonic otic vesicle at E9.5 stained with Pax2 (green) and TUJ1 (red) antibodies. Nuclei were counter stained using DAPI (blue). (C-C”) N33 cells were cultured under proliferative conditions and stained with Pax2 (green) and LSD1 (red) antibodies. Scale bars, 50 μm.

Histone methylation and demethylation are dynamic processes that activate or repress gene expression, leading to cellular differentiation [33]. To better understand the role of histone demethylation in Pax2-mediated gene regulation, we first examined the expression pattern of Pax2 and LSD1 in the mouse otocyst at embryonic day 9.5. We found that Pax2 was expressed specifically in the ventral part of the otic vesicle, whereas LSD1 was expressed throughout (Fig 1A). Pax2 and LSD1 were co-localized in cellular nuclei. Notably, Pax2 is down regulated in the pool of Tuj1+ neuroblasts that delaminate from the otic vesicle epithelium between E9 and E12, consistent with previous studies (E9.5 shown in Fig 1B; [34]). Thus, we wondered whether Pax2 is a negative regulator of otic neurogenesis. We also found that both Pax2 and LSD1 proteins are co-expressed in the nuclei of the majority, if not all, of N33 cells (Fig 1C). Moreover, we confirmed the presence of other chromatin complex proteins, namely mixed lineage leukemia 3 (MLL3) and Pax Transcription Activation Domain Interacting Protein (PTIP), in these cells (S1 Fig), consistent with previous studies where Pax2 was associated with PTIP in the MLL3-containing histone H3K4 methyltransferase complex in kidney progenitor cells [29].

The N33 cell line was derived from the ventral region of the otocyst in E10.5 immortomice^™^ and is thought to represent auditory neuroblasts [27]. Under differentiating conditions, N33 cells stop proliferating and give rise to beta-tubulin-expressing neuron-like cells [16]. They can also replace functional spiral ganglion cells in vitro [35], so we reasoned that they could be a suitable model system to study epigenetic changes associated with otic progenitor differentiation. The co-existence of histone methyl mark modifying proteins LSD1 and MLL3 suggests that there may be a fine balance between histone methylation and demethylation during the otic progenitor-to-neuroblast transition. Since Pax2 and LSD1 are co-expressed *in vivo* and *in vitro*, and both have a well-established involvement in chromatin modifying complexes, we hypothesized that Pax2 and LSD1 would interact in otic progenitor cells.

### Pax2 and LSD1 are novel interacting partners and associate with the NuRD complex

**Fig 2:**
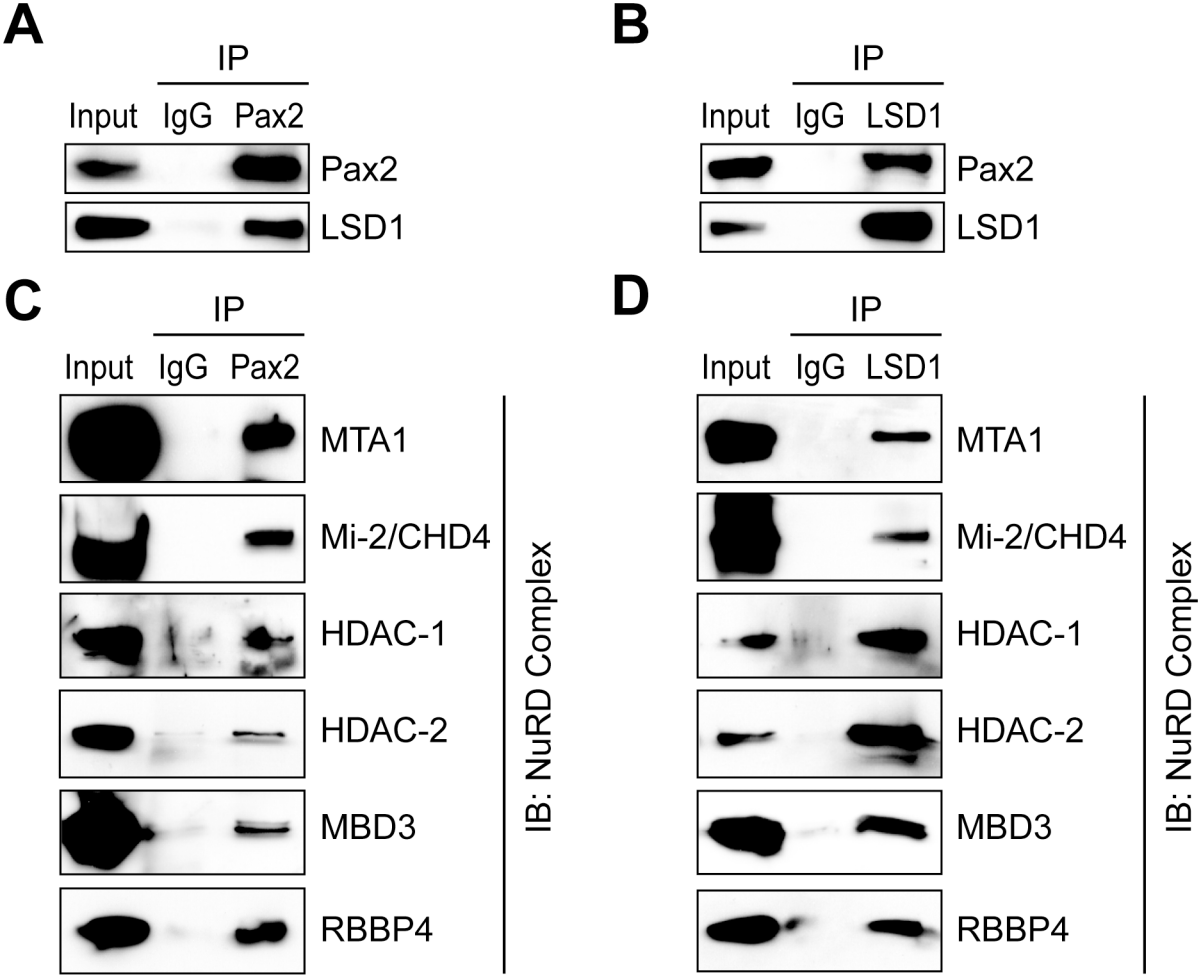
Pax2 interacts with LSD1 and is associated with the NuRD complex. (A, B) Physical interaction between endogenously expressed Pax2 and LSD1 proteins in N33 cells. Nuclear cell lysates were prepared from undifferentiated N33 cells and immunoprecipitations with either anti-LSD1 or anti-Pax2 antibody were performed. (C, D) Pax2 and LSD1 immunoprecipitated complexes were probed against various members of the NuRD complex as indicated. Immunoprecipitated complexes were resolved by SDS-PAGE followed by immunoblot analysis using indicated antibodies. Rabbit IgG was used as a negative control for non-specific immunoprecipitation. Cell lysates before co-immunoprecipitation were used as input controls.

Chromatin-modifying enzymes may need a gene-specific transcription factor to regulate histone modifications at the precise target promoter [36]. Pax2 has a unique spatio-temporal pattern of expression during inner ear development [37]. We performed co-immunoprecipitation to determine a possible interaction between endogenously expressed Pax2 and LSD1. Remarkably, we found that Pax2 interacts with LSD1 in undifferentiated N33 cells (Fig 2A and 2B). This previously unrecognized interaction between Pax2 and LSD1 led us to determine other LSD1-interacting proteins that can form a co-repressor protein complex for Pax2. Previous work has demonstrated that LSD1 has a dual role in transcriptional activation and repression during developmental gene activation or repression [33]. LSD1 regulates gene expression by modifying specific histone H3 methyl marks, which depend upon recruitment of LSD1 in different chromatin complexes, so we performed further immunoprecipitation assays to determine specific proteins associated with LSD1-Pax2 complexes. Our immunoprecipitation analyses revealed a specific enrichment of major components of the NuRD transcriptional repressor complex, such as metastasis-associated 1 (MTA1), chromodomain-helicase-DNA-binding protein 4 (CHD4, also known as Mi-2), HDAC1, HDAC2, methyl-binding domain protein 3 (MBD3) and the retinoblastoma binding protein 4 (RBBP4), in both Pax2 and LSD1 co-immunoprecipitates (Fig 2C and 2D). In contrast, we did not detect interactions of the CoREST and SIRT1 complexes with endogenous Pax2 (data not shown).

### Pax2 and the LSD1/NuRD complex occupy the Pax2 responsive element in N33 cells

**Fig 3:**
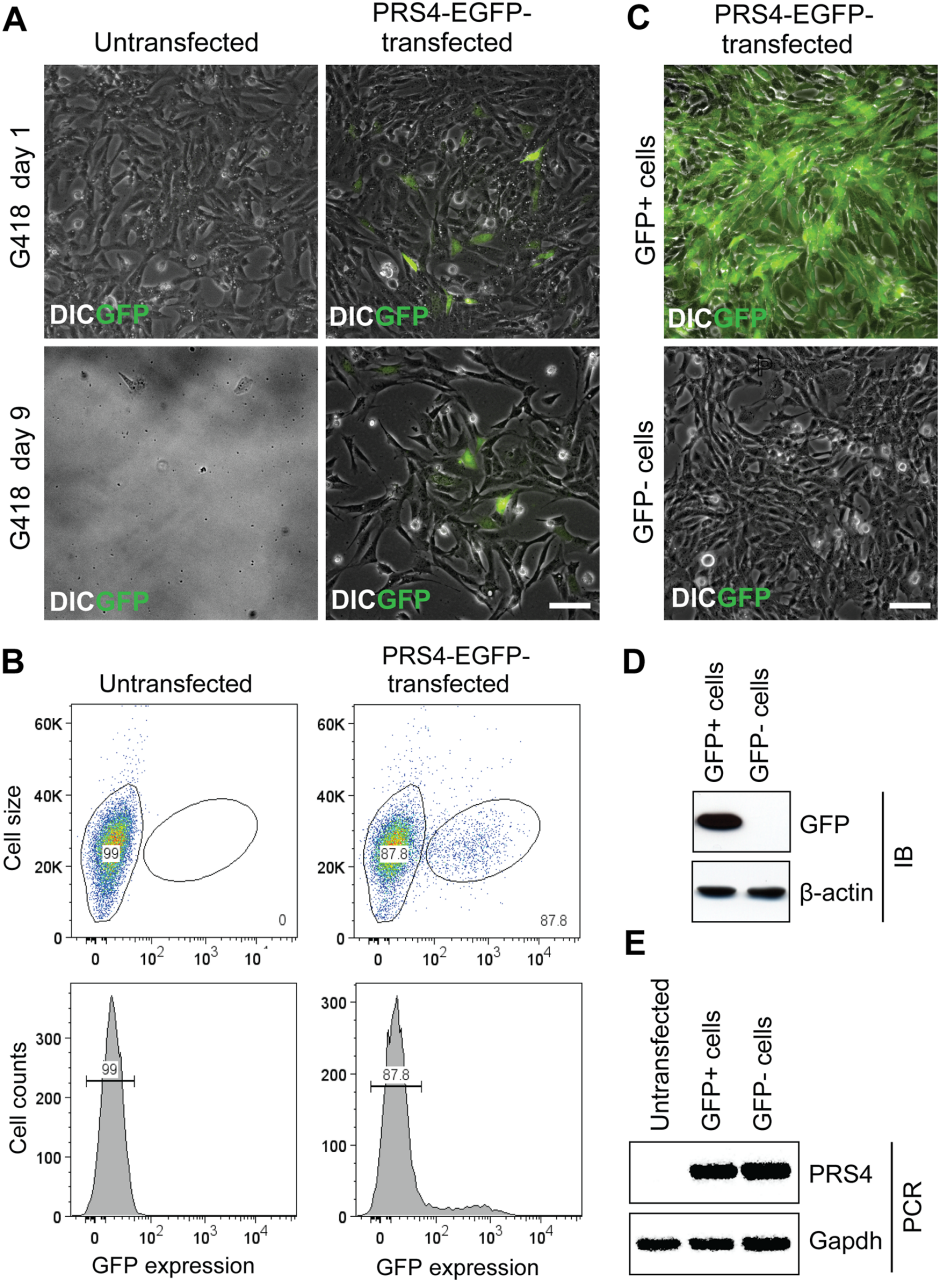
Generation and characterization of PRS4-EGFP N33 (PE) stable cells. (A) A linearized PRS4-EGFP construct was transfected into N33 cells. G418 antibiotic at 500 μg/ml was used as a negative selection for stable clones. The upper panel indicates cells after 24 hours of transfection. As indicated in the lower panel, almost all non-transformed cells died by day 9, but cells with stable incorporation of the PRS4-EGFP cassette (PE) survived. (B) FACS sorting of PE positive and negative cells based upon GFP expression. N33 cells were used as a negative control for gate setup during separation of PE negative cells. The lower panel in the figure shows histogram representation of the upper panel. (C) FACS sorted PE positive (PE + ve) and PE negative (PE—ve) cells were separately grown and analyzed for GFP expression by fluorescence microscopy. (D) GFP expression was determined by Western blot analysis using GFP antibodies. Upper panel in the Western blot shows the absence of GFP expression in the PE—ve cells. β-ACTIN protein was used as a loading control in the lower panel. (E) PCR analysis was performed to confirm the presence of the PE cassette integration in DNA isolated from PE negative cells using PRS4 sequence specific primers (upper panel). PCR primers designed in the promoter region of *Gapdh* were used as an equal loading control for PCR products (lower panel). Chromatin isolated from normal N33 or PE positive cells were used as negative and positive controls, respectively. Scale bars, 100 μm.

Based on our finding that Pax2 and LSD1 are associated with the NuRD transcriptional repressor complex, we wished to elucidate the mechanisms underlying Pax2-mediated gene expression. We analyzed 32 putative genes for the Pax2 DNA binding consensus sequence using transcription factor scan (TFscan) software, but our stringent analysis failed to determine exact similarity with the known Pax2 binding site. Since there is a lack of available information on direct target genes for Pax2 from *in vivo* studies, we decided to take advantage of the Pax2 responsive DNA binding element driving EGFP coding gene (PRS4-EGFP construct, [9]), to test DNA occupancy of the Pax2-interacting proteins we had identified. The PRS4-EGFP reporter construct contains five copies of the Pax2-responsive DNA binding element inserted upstream to the thymidine kinase (TK) minimal promoter, driving EGFP expression [29]. In addition, the PRS4-EGFP construct also carries the G418 antibiotic resistance gene for negative selection. During antibiotic selection to establish a N33 cell line stably expressing PRS4-EGFP, we noticed a mosaic of EGFP positive and negative cells (Fig 3A). We employed fluorescence-activated cell sorting (FACS) to isolate populations of EGFP-positive and -negative cells. The majority of the cells were (~90%) EGFP negative, whereas ~ 10% of the cells were EGFP positive (Fig 3B). When propagated under proliferation conditions, the EGFP-negative and -positive cells remained negative and positive, respectively, after a number of passages (Fig 3C). Western blot analysis further confirmed the presence and absence of EGFP expression in respective cell types (Fig 3D). However, the PRS4-EGFP construct was present in the EGFP-negative cells using a PCR primer pair flanking the PRS4 sequence (Fig 3E), suggesting that EGFP expression is silenced in these cells.

**Fig 4:**
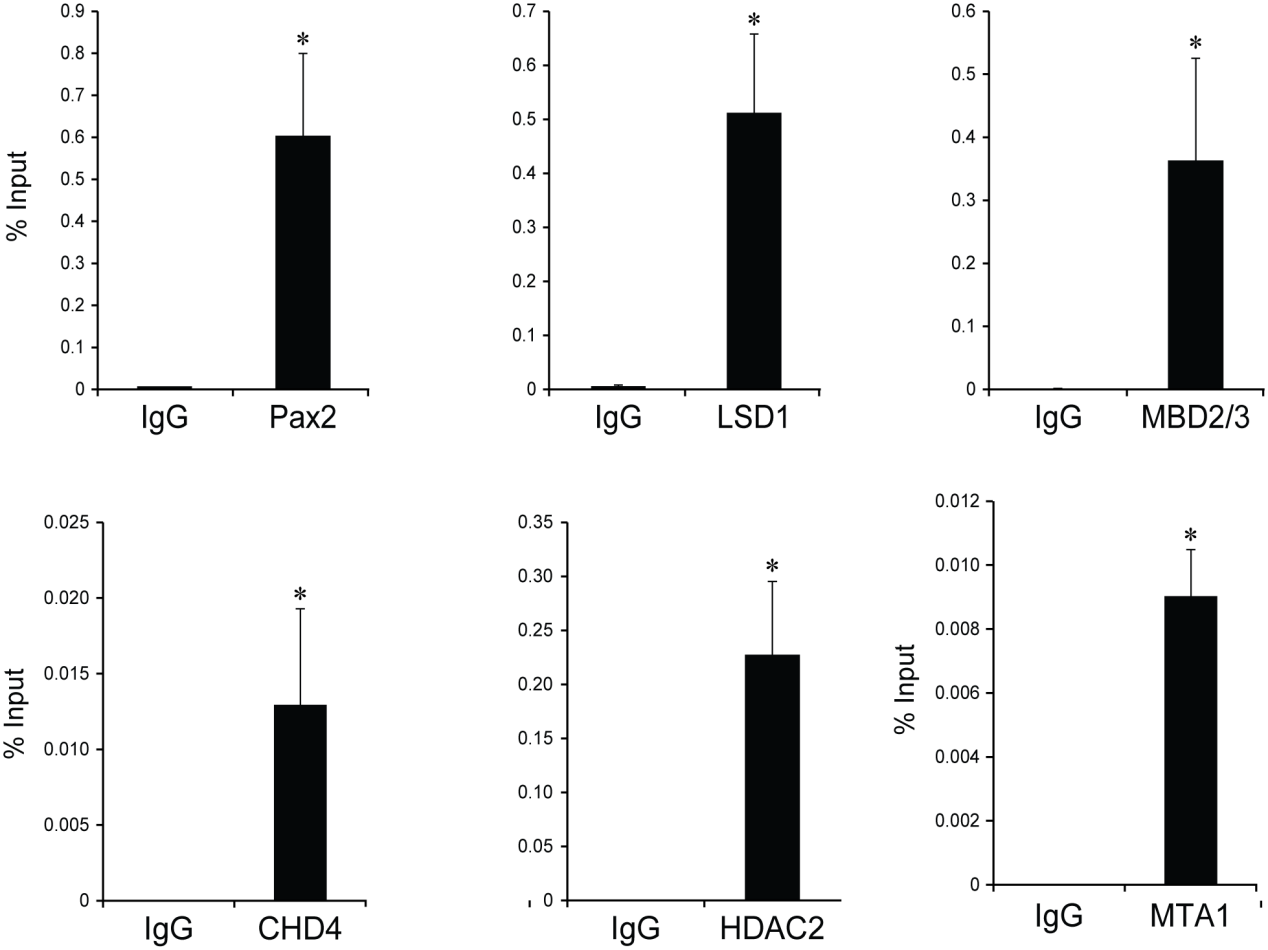
PAX2-LSD1/NuRD complexes are recruited on the Pax2 binding elements. ChIP experiments were performed on PE negative cells using a variety of antibodies as indicated in each graph. Primer pairs flanking to the Pax2 response element were used for real-time quantitative PCR and relative amounts of PCR products are expressed as a percent of input chromatin. Equal concentrations of rabbit or mouse IgG were used as negative controls for respective ChIP experiments. All values are expressed as the mean of 3 replicates; error bars indicate one standard deviation. Statistically significant differences are indicated (*P<0.05).

We hypothesized that Pax2 constitutively binds to the Pax2-responsive DNA elements in N33 cells and recruits the NuRD transcriptional repressor complex through its interaction with LSD1. To test this hypothesis, we performed chromatin immunoprecipitation using Pax2 and LSD1 antibodies (Fig 4). PCR primer pairs flanking the PRS4 region were used to determine the degree of enrichment relative to input chromatin. We found that Pax2 binds to the PRS4 sequence as expected. Furthermore, LSD1 and major components of the NuRD complex such as MDB2/3, CHD4, HDAC2 and MTA1 are enriched at the PRS4 sequence (Fig 4). As controls, we tested for possible enrichment of components of the CoREST complex, such as CoREST, CtBP1 and CtBP2, at the PRS4 site; however, their recruitment was significantly lower than the enrichment of the NuRD complex protein, RBBP4 (data not shown).

### Blockage of LSD1/NuRD complex activity de-repress *EGFP* expression in PE negative cells

**Fig 5:**
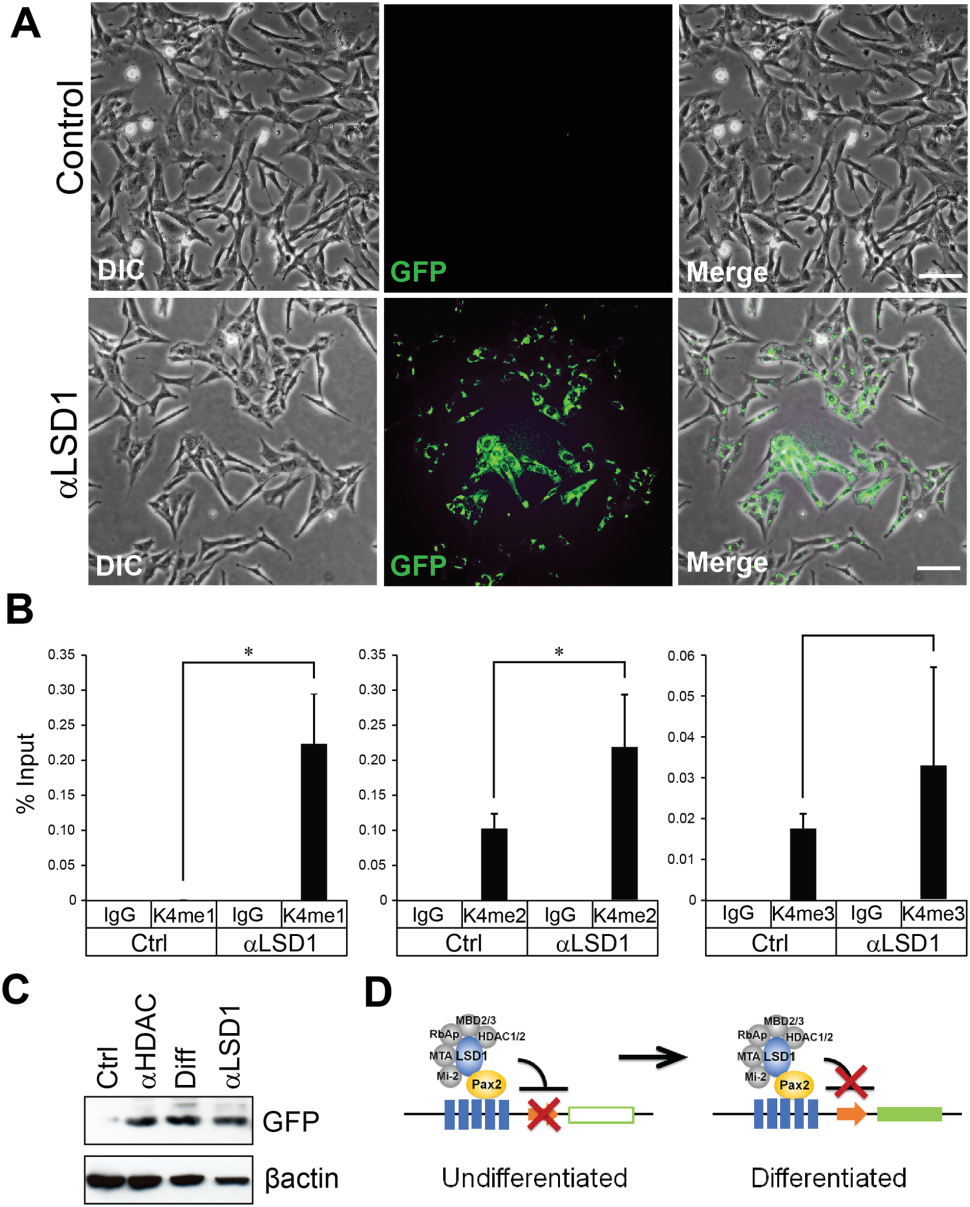
Blockade of LSD1/NuRD activity derepresses *Egfp* expression in PE negative cells. (A) PE negative cells were treated with DMSO (control) or 0.25 mM TCP for 48 hours and DIC and fluorescence images were captured. (B) PE negative cells were treated with DMSO (control) or TCP (48 hours) to inhibit LSD1. ChIP analyses were performed using H3K4 me1, me2 or me3 methyl marks specific antibodies as indicated. Rabbit IgG was used as a negative control. The immunoprecipitated chromatin was quantified by quantitative PCR using primers flanking to the Pax2 response element. The relative amounts of PCR products are expressed as a percent of input chromatin. All values are expressed as the mean of 3 replicates; error bars are one standard deviation. Statistically significant differences are indicated (*P<0.05). (C) PE negative cells were treated with DMSO (control), TSA, and TCP or alternatively differentiated by temperature induction for 48 hours followed by Western blot analysis of GFP protein expression. β-ACTIN was used as a protein loading control. (D) Schematic figure showing how GFP expression is activated in PE N33 cells upon differentiation.

The NuRD complex contains two major enzymatic components, a histone demethylase, LSD1 and the histone deacetylases, HDAC1 and HDAC2. If we block the enzymatic activity of the NuRD repressive complex in EGFP-negative, N33 cells using specific inhibitors, it should result in the expression of downstream *EGFP* gene in these cells. To block LSD1 demethylation activity, we used the well characterized LSD1 inhibitor TCP. Initially we performed dose response analyses and identified an optimal concentration of 0.25 mM of TCP for N33 cells (data not shown). EGFP negative cells were treated with TCP for 48 h and analyzed for EGFP expression by fluorescence microscopy. We observed an increase in EGFP expression with the inhibition of LSD1 activity, which we further confirmed by western blot analysis (Fig 5A and 5E). LSD1 is implicated in cell proliferation [38], and as expected, inhibition of LSD1 activity in N33 cells resulted in reduced proliferation as evident by the lower cell density at 48 h when compared to untreated control cells (Fig 5A). Histone H3K4 methyl marks are associated with active gene transcription [39]. LSD1 can demethylate mono- and di-methylation of histone H3 on lysine 4 (H3K4me1 and me2) marks but not tri-methylation of histone H3 on lysine 4 (H3Kme3) [40]. The elevated EGFP expression upon LSD1 inhibition was accompanied by an increase in histone H3K4me1 and me2 active methyl marks, but not in the H3K4me3 mark. The increase in H3K4 methyl marks on PRS4 sites were validated by ChIP assays, using specific antibodies against histone H3K4me1/me2/me3 methyl marks (Fig 5B–5D).

HDACs are also components of the NuRD complex and participate in gene silencing by removing acetyl marks from histones. Therefore, inhibition of HDACs results in an increase in histone acetylation. Acetylated histones that are released from condensed chromatin facilitate gene transcription. HDAC1/2 deacetylase activity could be targeted using the widely used HDAC inhibitor TSA. HDAC inhibition is also associated with decreased cell proliferation [36], for that reason we seeded cells at a higher cell density for TSA treatment. EGFP-negative N33 cells treated with TSA for 48 h started to express EGFP, as demonstrated by both western blot and immunofluorescence analysis (S2A and S2B Fig). Collectively, our data suggest that the Pax2/LSD1/NuRD complex constitutively occupies the Pax2 responsive gene promoter and represses transcription in N33 cells.

### Pax2/LSD1/NuRD complex maintains otic progenitor status

Neurogenesis in the inner ear depends upon the function of the proneural basic helix-loop-helix (bHLH) transcription factors Neurogenin1 and NeuroD1 [41, 42]. Since Pax2 expression is downregulated in delaminating neuroblasts (Fig 1B), we hypothesized that the Pax2/LSD1/NuRD complex may maintain an otic progenitor pool by preventing neural differentiation. To test this hypothesis, N33 cells were subjected to differentiation. Consistent with our hypothesis, we observed a reduced expression of *Pax2* following differentiation (S3 Fig). Our quantitative PCR analysis also revealed robust increases in the expression of key neuronal genes such as *NeuroD1* and *Ngn1*, along with the migrating neuroblast marker gene *Phox2b* (S3 Fig). In addition, inner ear sensory neuron marker genes such as *Brn3a* and *Tlx3* were up-regulated. By contrast, the proliferation-associated gene *c-Myc* was significantly down regulated upon differentiation. The expression of DNA-directed RNA polymerase II subunit RPB1 encoding gene *Polr2a* remained unchanged.

**Fig 6:**
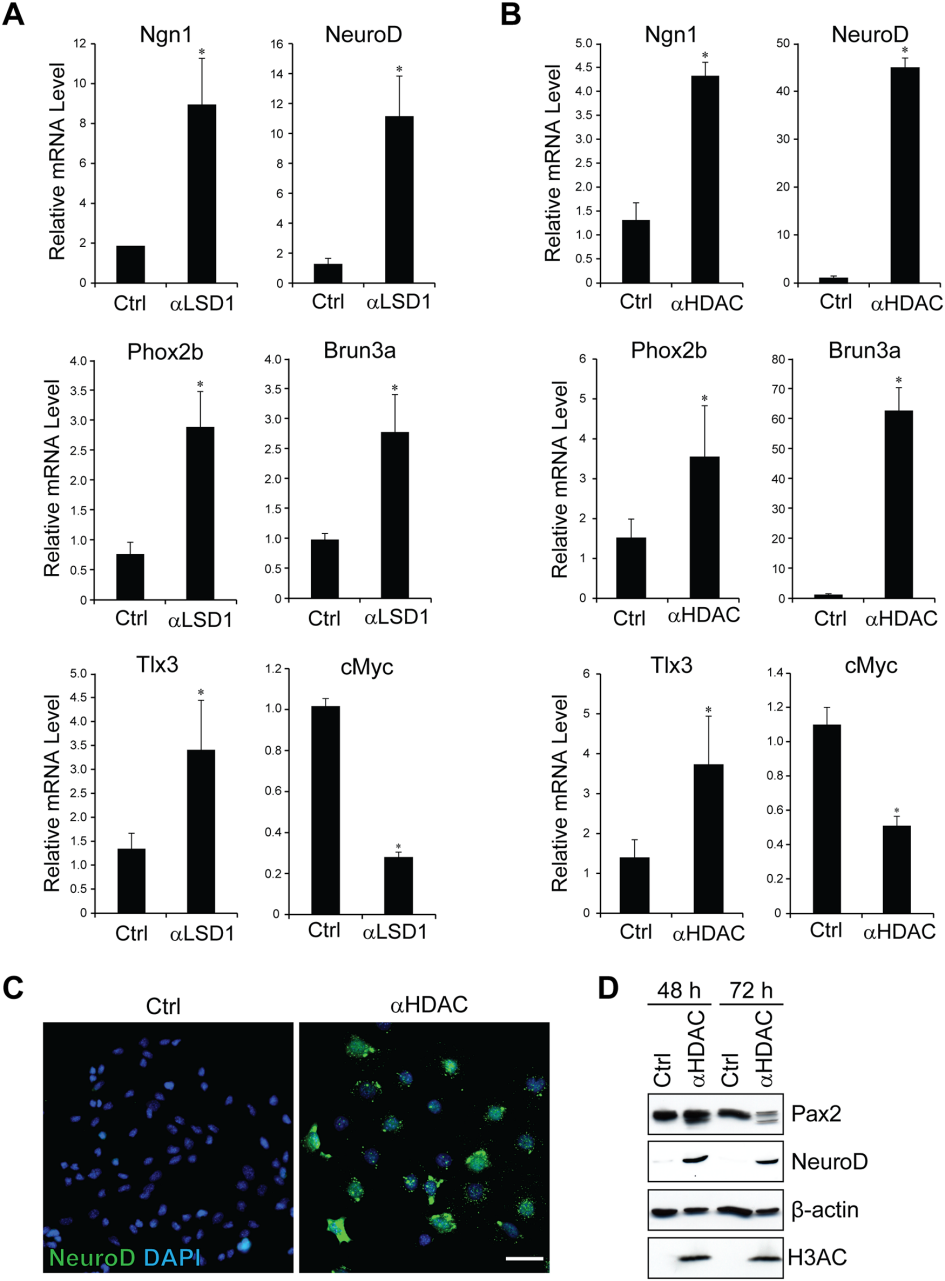
PAX2/LSD1/NuRD complex suppresses neuronal differentiation of N33 cells. (A, B) N33 cells were cultured under proliferative conditions. Cells were washed once the following day with PBS and treated with DMSO (control) or TSA, LSD1-C12 for 48 hours in the absence of gamma interferon. Quantitative real-time RT-PCR analysis was used to examine changes in the mRNA expression of the proneural markers *NeuroD* and *Ngn1*, the migrating neuroblast marker *Phox2b* and the inner ear sensory neural markers *Tlx3* and *Brn3a*. Expression levels were normalized to those of the housekeeping gene *L27*. All values are expressed as the mean of 3 replicates; error bars indicate one standard deviation. Statistically significant differences are indicated (*P<0.05). (C) N33 cells were cultured and treated under similar conditions to those described in A and B. Cells were washed once with PBS and fixed in 4% PFA at room temperature. Immunostaining was performed for analysis of NeuroD protein expression. Nuclei were counter stained using DAPI (blue). Scale bar, 100 μm. (D) N33 cells were treated with DMSO control or TSA for 48 or 72 hours. Western blot analysis was performed to confirm a significant up-regulation of NeuroD. β-ACTIN was used as a loading control. Histone 3 Acetyl mark specific (Ac-H3) antibody was used to show an increase in acetylation, as a positive control to TSA treatment.

As a proof of concept, we treated undifferentiated N33 cells with either a potent LSD1 inhibitor or HDAC inhibitor for 48 h. Our results revealed that inhibition of LSD1 or HDAC resulted in a significant increase in the expression level of *NeuroD1*, *Ngn1*, *Phox2b*, *Brn3a* and *Tlx3* (Fig 6A and 6B), consistent with the results after temperature-induced cell differentiation. To confirm that the gene expression data may signify a change in protein level, we examined the expression of NeuroD1 protein by immunofluorescence staining of N33 cells treated with either an HDAC inhibitor or DMSO (Fig 6C). Remarkably, the number of NeuroD1-positive cells appeared to increase by HDAC inhibition, which was confirmed with Western blot analysis (Fig 6D). As a positive control, we used a Histone 3 Acetyl mark (Ac-H3) specific antibody in our Western blot analysis to show an increase in acetylation of histone due to HDAC inhibition. These data altogether suggest that Pax2 acts as a negative regulator of neuronal differentiation by interacting with the LSD1/NuRD complex.

To test the effects of LSD1 inhibition on otic progenitor cells, we treated mouse embryonic stem cell-derived inner ear organoids with the small molecule inhibitor for 48 hrs starting at day 8 of differentiation (S4 Fig). This treatment resulted in a significant increase in Brn3A/TUJ1-positive sensory neuron-like cells, further confirming that LSD1 functions to suppress neuronal differentiation from otic progenitors *in vitro*.

## Discussion

LSD1 is a flavin-dependent monoamine oxidase, which can demethylate mono- and di-methylated histone 3 at lysine 4 (H3K4) [16, 18, 19]. This enzyme has been shown to play critical roles in embryogenesis and tissue-specific differentiation [33]. In the present study, we have identified LSD1 as a novel interacting partner of Pax2 in otic progenitors. We found that LSD1 is colocalized with Pax2 in the ventral region of the otic vesicle, from which all sensory cell types in the inner ear arise. In the otic neuroblast cell line N33, Pax2 and LSD1 interact with the NuRD co-repressor complex, which contains histone deacetylases HDAC1 and HDAC2, and together form a Pax2/LSD1/NuRD protein complex. Components of this protein complex constitutively occupy Pax2-binding loci in N33 cells. Inhibition of LSD1 activity by a small molecule inhibitor results in an increase in the expression level of several prosensory genes and proteins in both N33 cell line and stem cell-derived otic progenitors through increased methylation of H3K4. These results suggest LSD1 as a previously unrecognized modulator for Pax2-mediated sensory differentiation in the inner ear.

**Fig 7:**
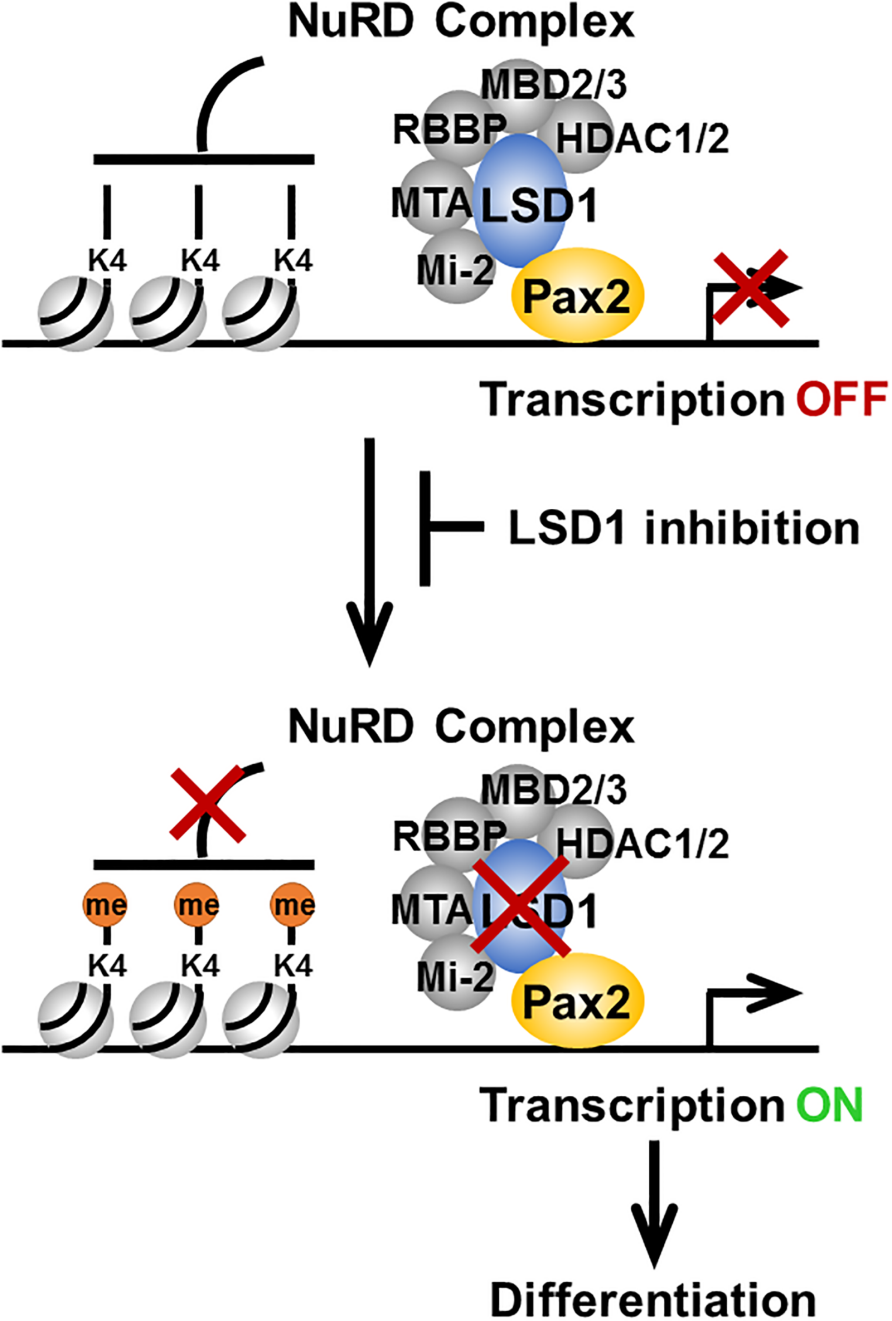
A model of LSD1-mediated epigenetic gene regulation in otic progenitors. Schematic illustration of the association of LSD1 with Pax2 and the NuRD repressor complex and recruitment of this complex onto Pax2 responsive genes leading to transcriptional repression in otic progenitors prior to differentiation. Inhibition of LSD activity results in an increase in H3K4 methylation, thereby triggering transcriptional activation of Pax2 downstream target genes. Activation of prosensory genes leads to neurosensory fate specification and differentiation in the inner ear.

During inner ear development, mechanosensitive hair cells, supporting cells and sensory neurons are derived from multipotent progenitors in the otic vesicle [41, 43]. Proper cell fate specification of these cell types is believed to require a precisely orchestrated cascade of spatial and temporal gene expression [43]. Pax2 is one of the transcription factors known for its critical role in inner ear development in mice and humans [23–26, 44, 45]. Thus, identifying key components of the Pax2 transcriptional regulatory network would provide clues for better understanding the molecular mechanisms underlying cellular differentiation and cell fate specification in the inner ear. Although LSD1 promotes transcriptional activation or repression in a context-depending manner, the LSD1/NuRD complex is usually associated with transcriptional repression [19, 46, 47]. Consistent with this idea, our present data show a lack of the Pax2-responsive element activation in untreated N33 cells and activation of the reporter gene expression through the Pax2-responsive element after treatment with a small molecule LSD1 inhibitor. Additionally, blocking LSD1 activity also increases the expression of genes related to the differentiation of spiral ganglion neurons in otic progenitor cells (Fig 6A–6C). Based on these results, we propose a model in which the LSD1/NuRD complex maintains otic progenitors in an uncommitted state and controls the timing and extent of neurogenesis in the developing inner ear (Fig 7). In multipotent otic progenitors, the LSD1/NuRD complex is recruited onto the promoters and/or enhancers of differentiation genes by Pax2, resulting in deacetylation of H3 and demethylation of H3K4, which are associated with transcriptional inactivation [48]. Once progenitors enter the differentiated phase, methylation of H3K4 and acetylation of H3 occur, leading to transcriptional activation of Pax2 downstream target genes.

Epigenetic regulation of the differentiation of spiral ganglion neurons remains poorly understood, but we previously showed that histone acetylation via CREB-binding protein (CBP) may be involved in this process [49]. Given that histone deacetylation by HDACs plays a pivotal role in both early neural specification and late neurogenesis during brain development [50–52], it is conceivable to assume that a similar mechanism might be involved in the differentiation of spiral ganglion neurons. In support of this idea, we found that a HDAC inhibitor promotes upregulation of several marker genes and proteins associated with developing spiral ganglion neurons in otic neuroblast N33 cells through the accumulation of acetylated histone H3 (Fig 6C and 6D, S2 Fig). We observed similar, albeit lesser, effects with a LSD1 inhibitor on spiral ganglion neuron-specific genes and confirmed that these effects are mediated through increased histone methylation of H3K4 (Figs 5B and 6A). These findings suggest that histone demethylation and deacetylation comprise a dual lock for otic progenitor differentiation and that suppressing either LSD1 or HDAC activity is sufficient for triggering transactivation of genes associated with neurogenesis in the inner ear. However, since inhibition of LSD1 leads specifically to methylation of H3K4 at Pax2-binding loci, this treatment may likely up-regulate a specific set of genes in contrast with HDAC inhibition, which results in widespread and uncontrolled acetylation of chromatin and consequently aberrant gene activation. It should be noted that we were not able to test the effects of LSD1 or HDAC inhibition on hair cell differentiation since we used the otic neuroblast cell line as a cellular model. Nevertheless, it will be tempting to explore the possibility of using an LSD1 inhibitor as a potential therapeutic target for promoting sensory regeneration in the inner ear. Future investigation should include a more direct approach to delete or to silence the genes encoding HDACs or LSD1 by gene knockdown and knockout *in vitro* and *in vivo* with an ultimate goal of validating the epigenetic mechanisms that we propose for neurogenesis of spiral ganglion cells.

## Supporting information

S1 Fig Constitutive expression of MLL3 and PTIP in N33 cells. N33 cells were cultured in proliferative condition and stained with Pax2 (green) and MLL3 or PTIP (red) antibodies. Scale bar, 50 μm.
(TIF)

S2 Fig HDAC inhibition leads to PAX-responsive reporter expression in N33 cells. (A) DIC and fluorescence merged images of PE negative cells treated with either DMSO (control) or TSA for HDAC inhibition for 48 hours. Scale bars, 100 μm. (B) PE negative cells were treated with DMSO (control), TSA for 48 hours followed by Western blot analysis of GFP and Pax2 expression. β-actin was used as a loading control whereas Ac-H3 was used as a positive control for TSA treatment.
(TIF)

S3 Fig Changes in mRNA expression in VT-N33 cells after differentiation. Quantitative real-time RT-PCR analysis was used to examine changes in the mRNA expression of proneural markers (*NeuroD* and *Ngn1*), a migrating neuroblast marker (*Phox2b*), inner ear sensory neural markers (*Tlx3*, *Brn3a*) and an otic progenitor marker (*Pax2*). *cMyc* and *Polr2a* serve as a cell proliferation marker and an RNA polymerase II marker, respectively. Expression levels were normalized to those of the housekeeping gene *L27*. All values are expressed as the mean of 3 replicates; error bars indicate one standard deviation. Statistically significant differences are indicated (*P<0.05).
(TIF)

S4 Fig LSD1 inhibition leads to an increase in sensory neuron-like cells in inner ear organoids. (A, B) Representative cross sections of inner ear organoids treated with DMSO (A) or LSD1-C12 (B) for 48 hrs. The samples were stained for Brn3a (green) and TUJ1 (red). Scale bar, 50 μm. (C) Quantitative comparison of the number of Brn3a/TUJ1-positive cells per section between DMSO verses LSD1-C12 treated organoids. We observed a significant increase in Brn3a+ cells in day 20 organoids treated with the LSD1 inhibitor (29.0±1.89) when compared with that of control (14.5±0.89) organoids (p<0.001). Brn3a+/Tuj1+ cells located within a distance of 4x radius of the otic vesicle were counted. n = 10 biological samples from 4–5 independent experiments.
(TIF)

S1 File Full-scan immunoblotting images. (PDF)
