# Supplementary figures and images for: The histone demethylase LSD1 regulates inner ear progenitor differentiation through interactions with Pax2 and the NuRD repressor complex
Source: PLoS One. 2018 Jan 25;13(1):e0191689. doi: 10.1371/journal.pone.0191689 (PMC5784988; doi:10.1371/journal.pone.0191689)

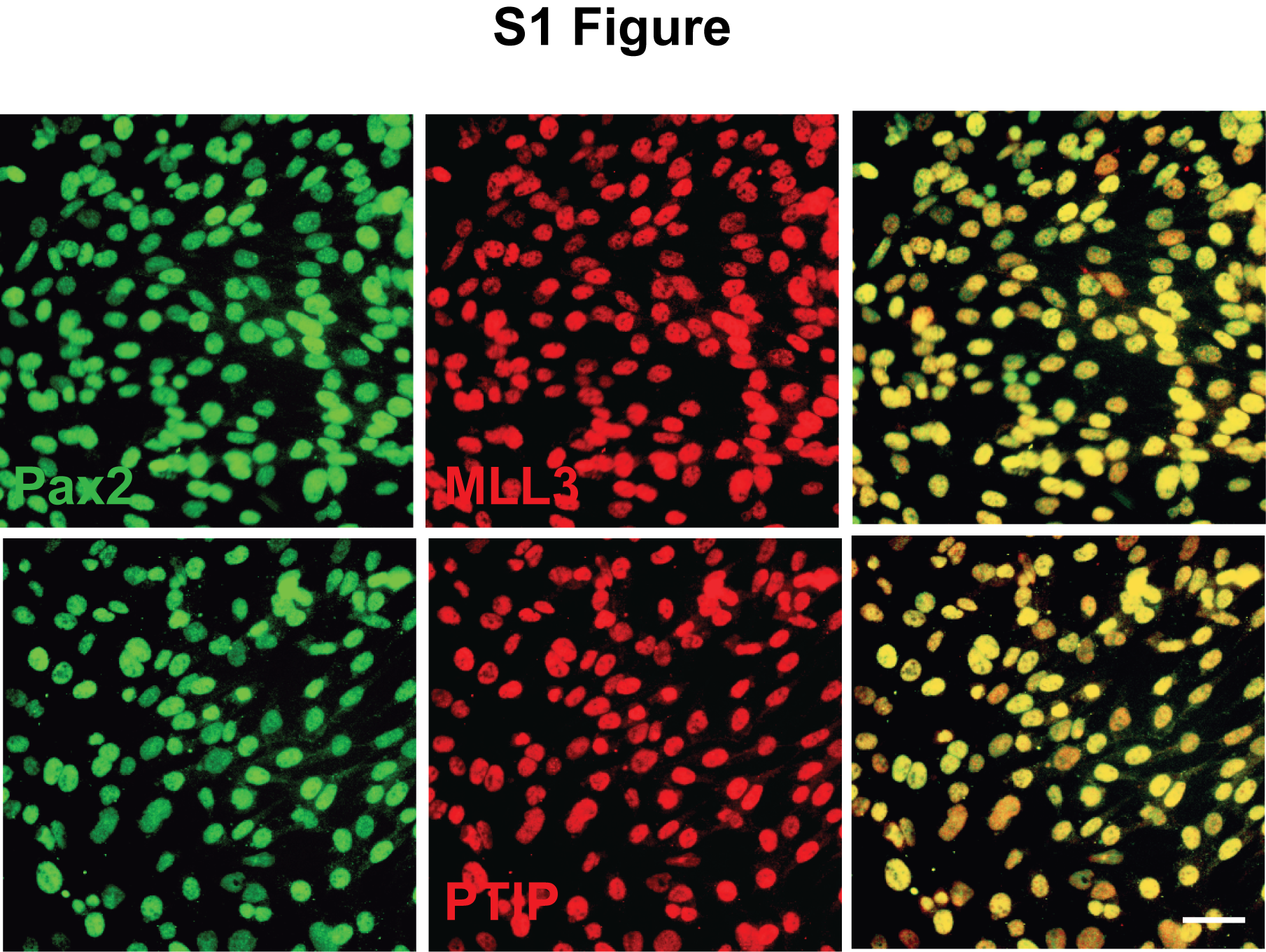

Supplement: S1 Fig — N33 cells were cultured in proliferative condition and stained with Pax2 (green) and MLL3 or PTIP (red) antibodies. Scale bar, 50 μm. (TIF) [file pone.0191689.s001.tif]

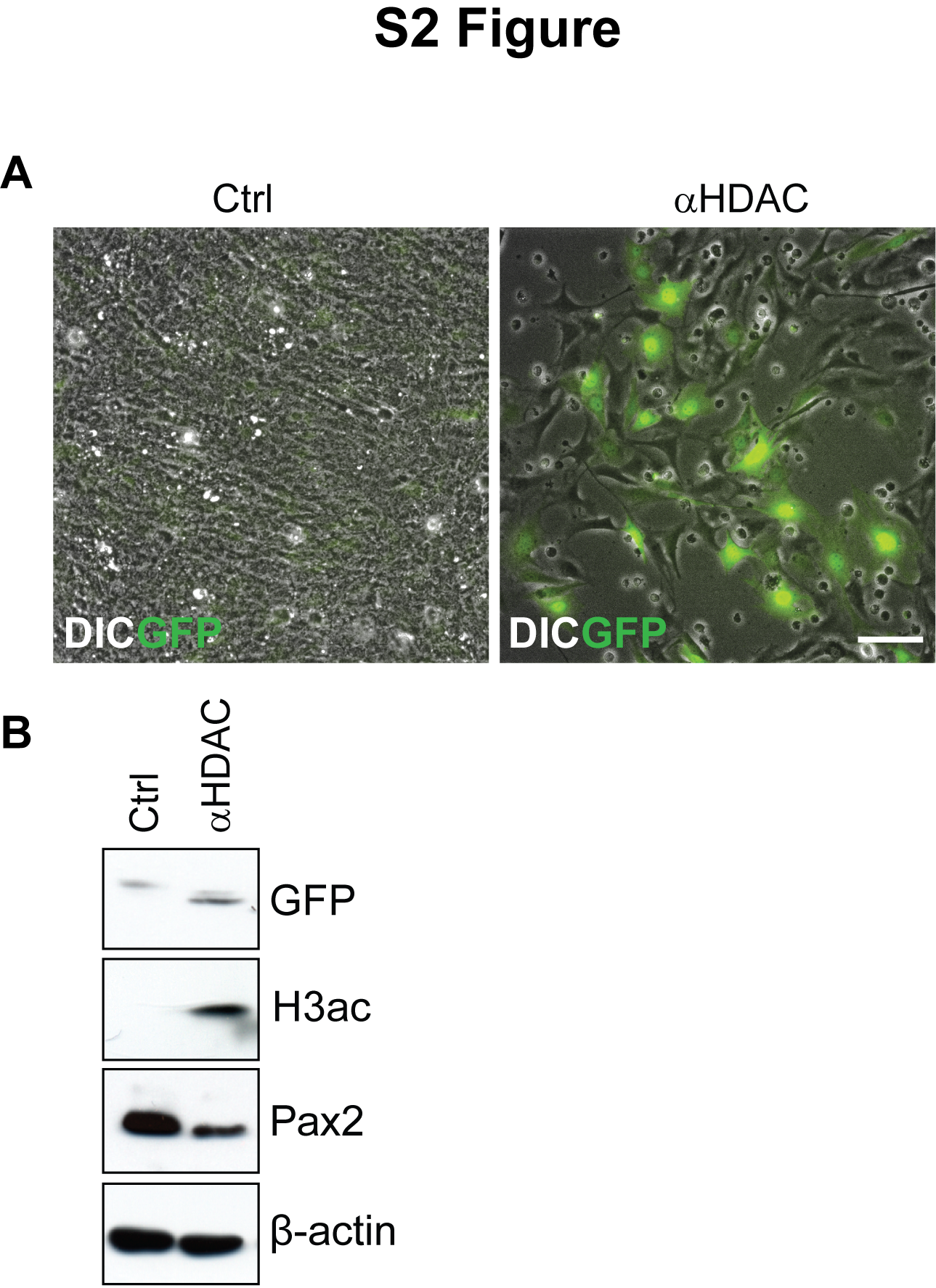

Supplement: S2 Fig — (A) DIC and fluorescence merged images of PE negative cells treated with either DMSO (control) or TSA for HDAC inhibition for 48 hours. Scale bars, 100 μm. (B) PE negative cells were treated with DMSO (control), TSA for 48 hours followed by Western blot analysis of GFP and Pax2 expression. β-actin was used as a loading control whereas Ac-H3 was used as a positive control for TSA treatment. (TIF) [file pone.0191689.s002.tif]

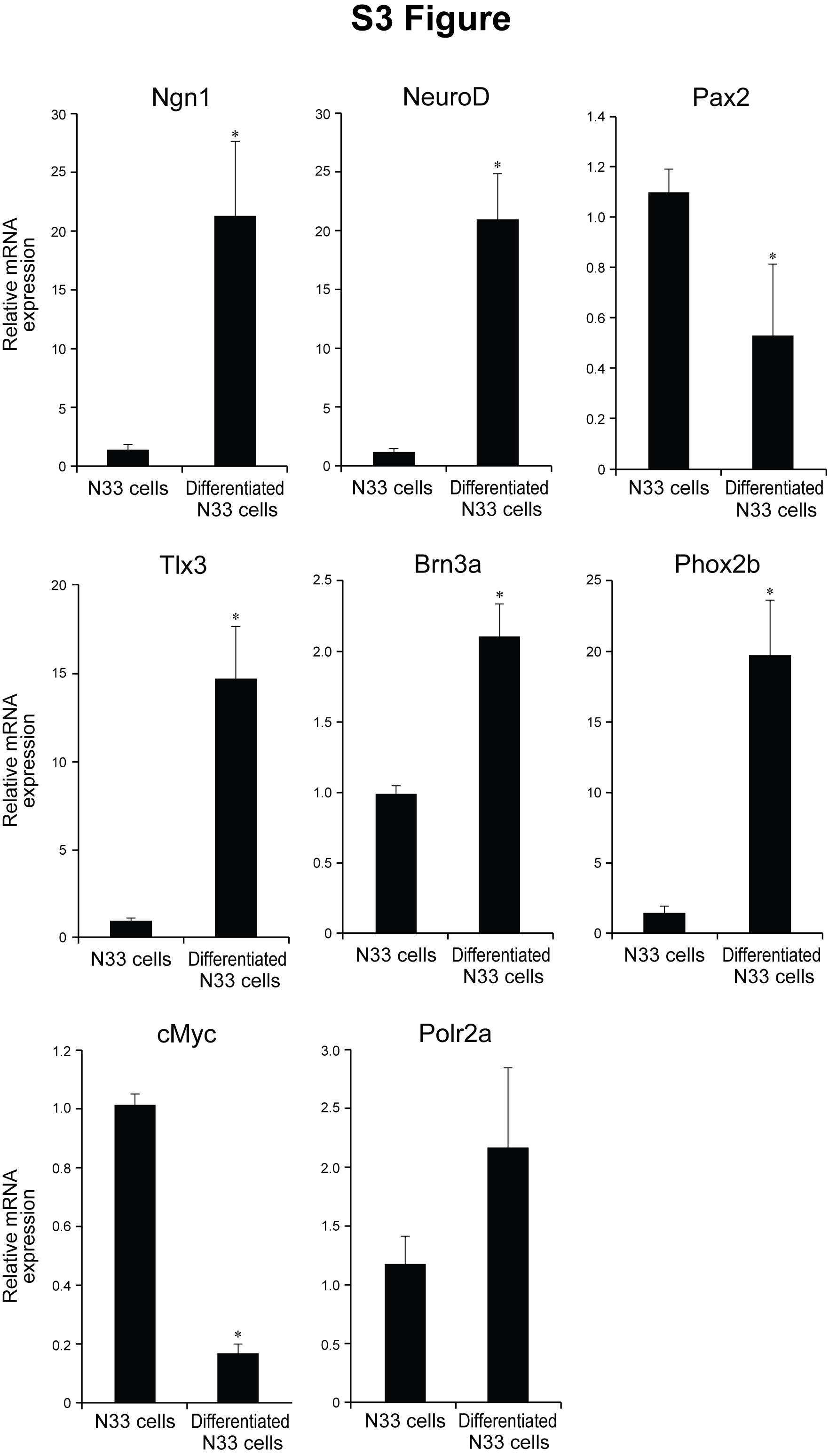

Supplement: S3 Fig — Quantitative real-time RT-PCR analysis was used to examine changes in the mRNA expression of proneural markers (NeuroD and Ngn1), a migrating neuroblast marker (Phox2b), inner ear sensory neural markers (Tlx3, Brn3a) and an otic progenitor marker (Pax2). cMyc and Polr2a serve as a cell proliferation marker and an RNA polymerase II marker, respectively. Expression levels were normalized to those of the housekeeping gene L27. All values are expressed as the mean of 3 replicates; error bars indicate one standard deviation. Statistically significant differences are indicated (*P<0.05). (TIF) [file pone.0191689.s003.tif]

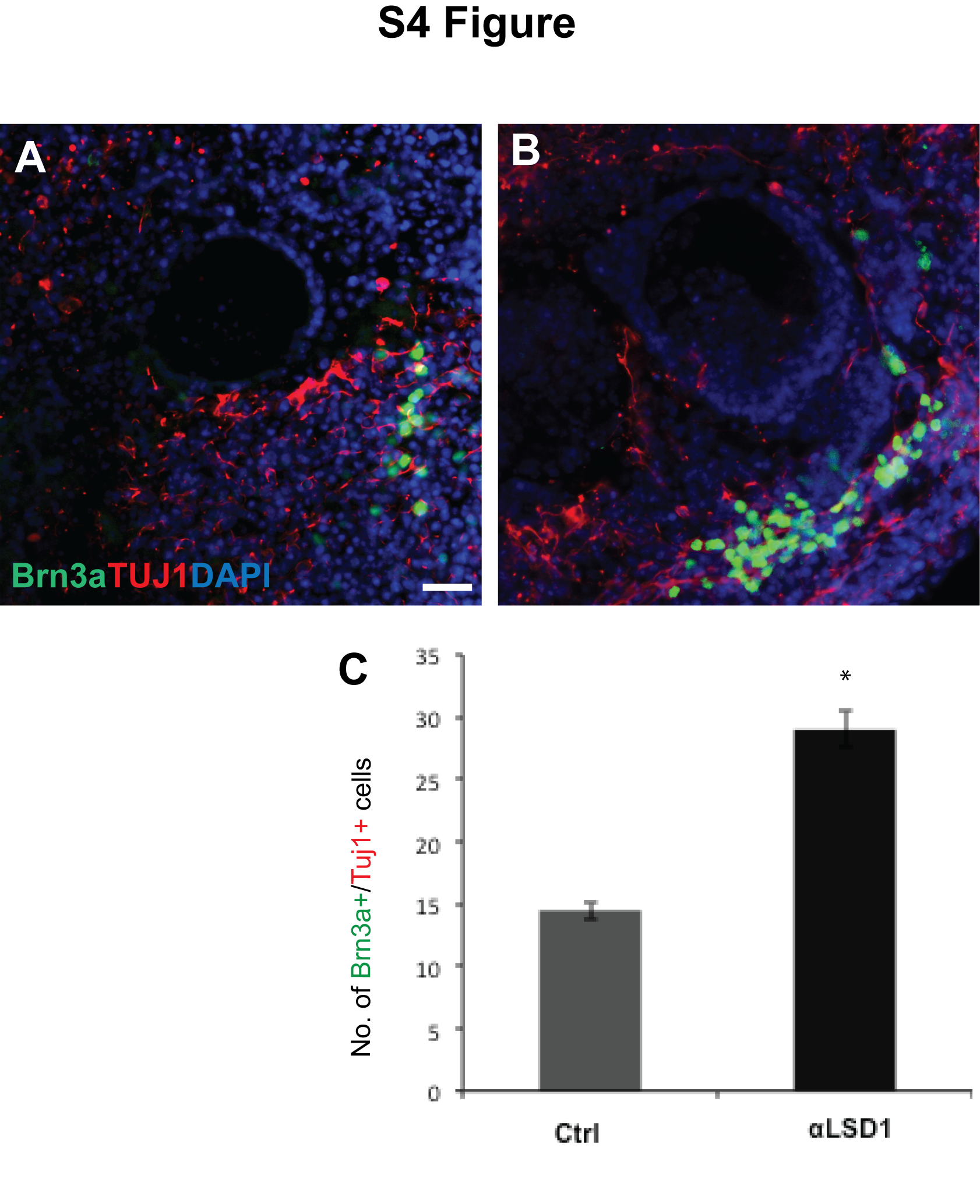

Supplement: S4 Fig — (A, B) Representative cross sections of inner ear organoids treated with DMSO (A) or LSD1-C12 (B) for 48 hrs. The samples were stained for Brn3a (green) and TUJ1 (red). Scale bar, 50 μm. (C) Quantitative comparison of the number of Brn3a/TUJ1-positive cells per section between DMSO verses LSD1-C12 treated organoids. We observed a significant increase in Brn3a+ cells in day 20 organoids treated with the LSD1 inhibitor (29.0±1.89) when compared with that of control (14.5±0.89) organoids (p<0.001). Brn3a+/Tuj1+ cells located within a distance of 4x radius of the otic vesicle were counted. n = 10 biological samples from 4–5 independent experiments. (TIF) [file pone.0191689.s004.tif]

**Figure 2A**

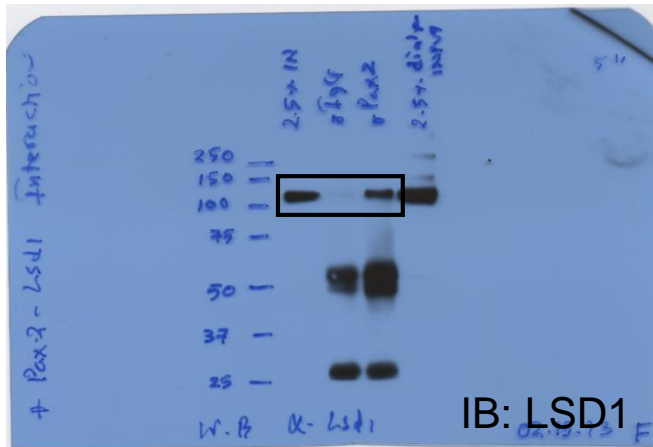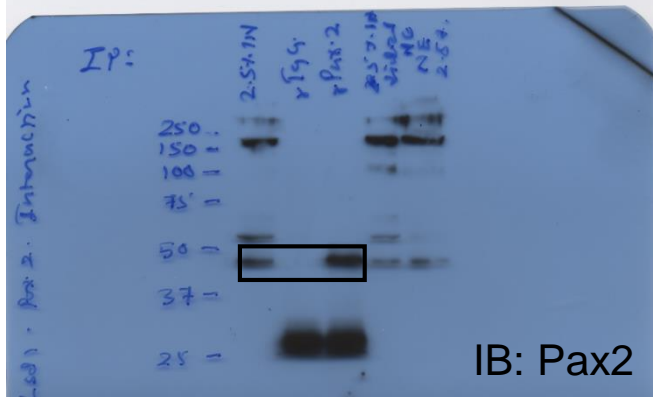

**Figure 2B**

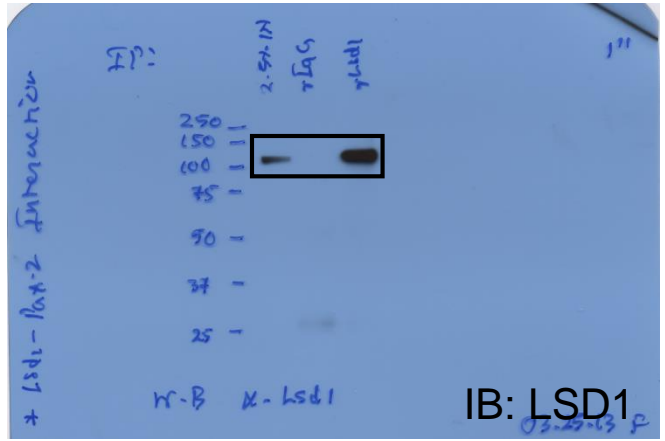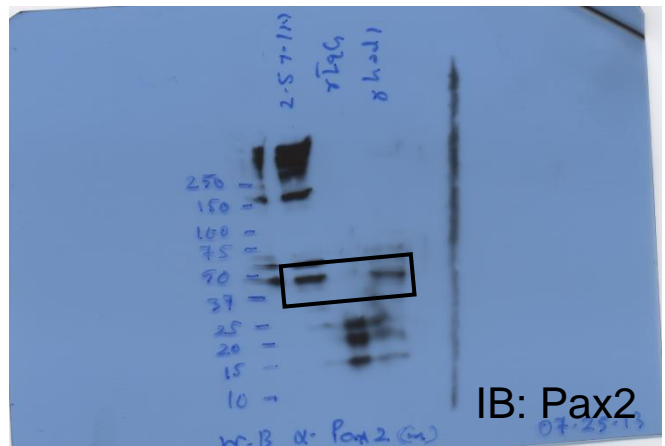

Figure 2C

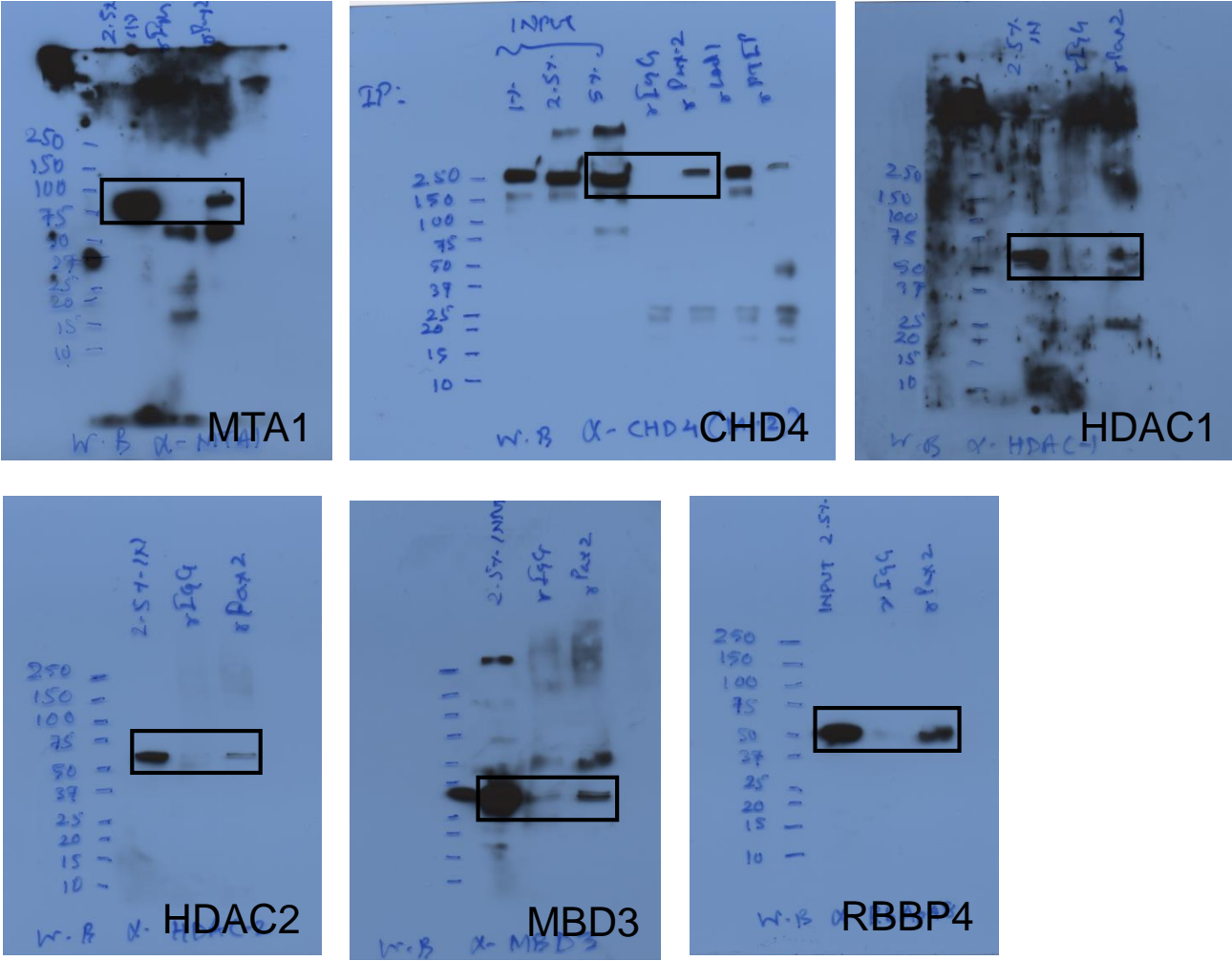

Figure 2D

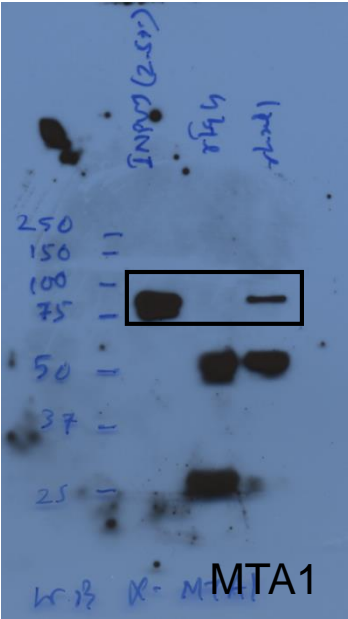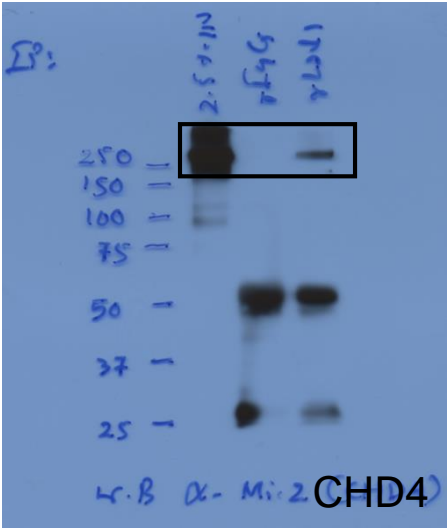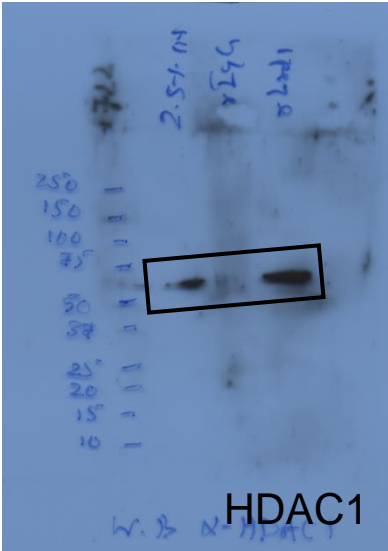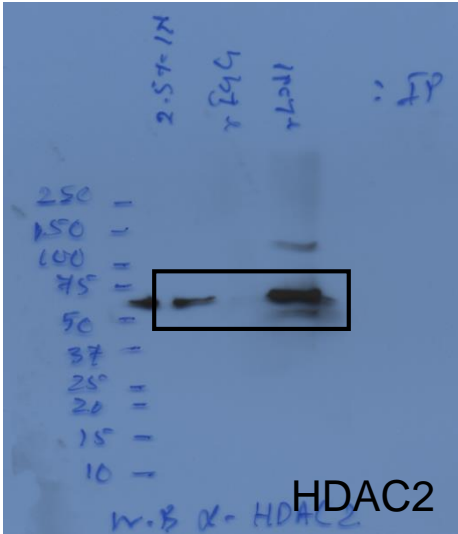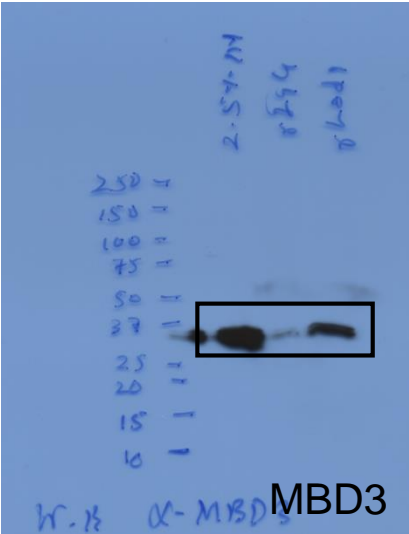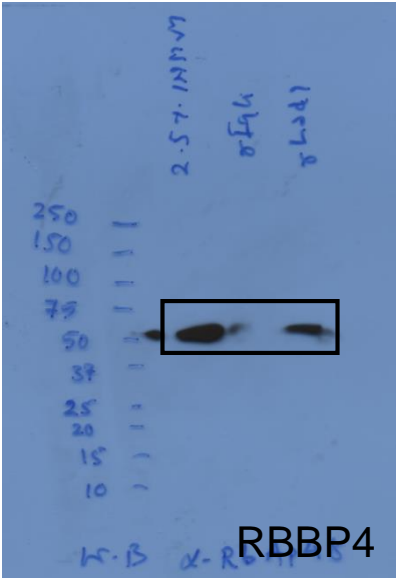

Figure 5C

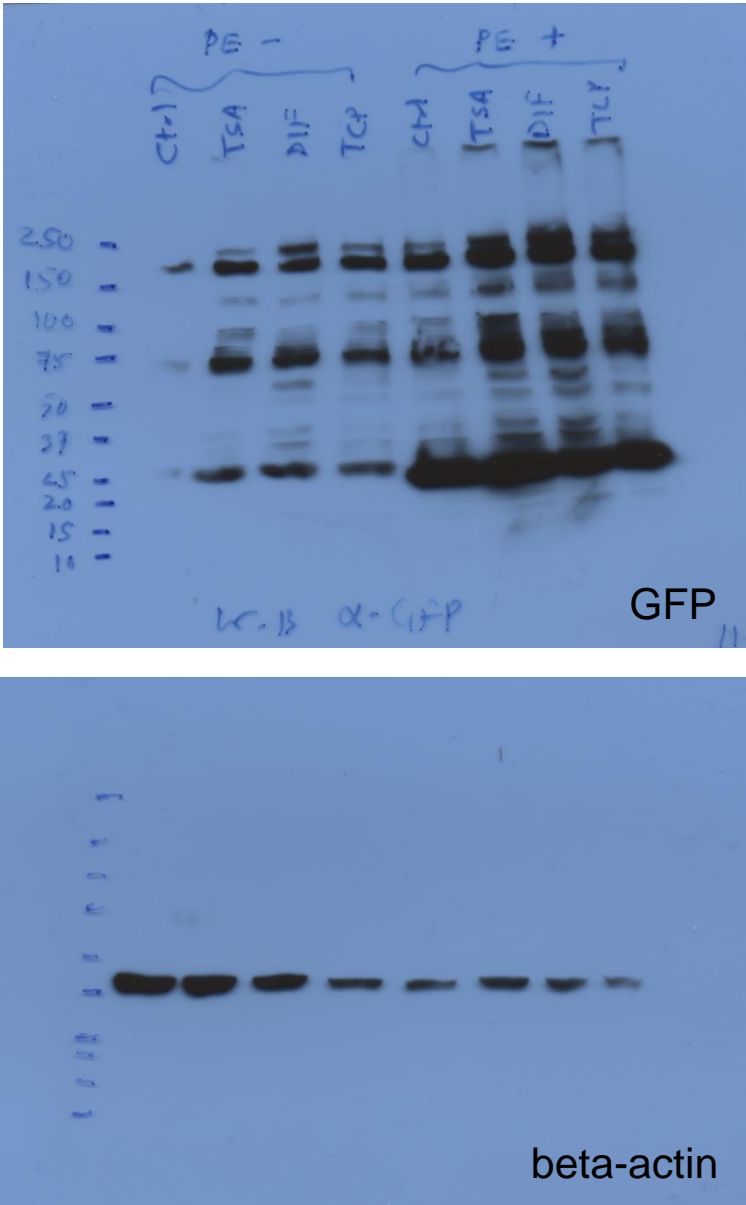

Figure 6D

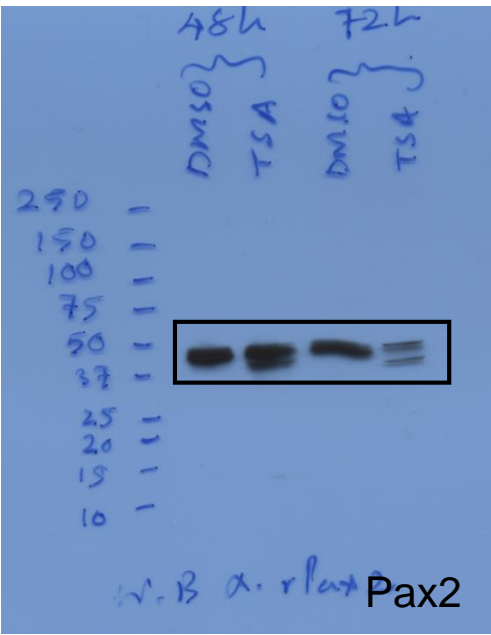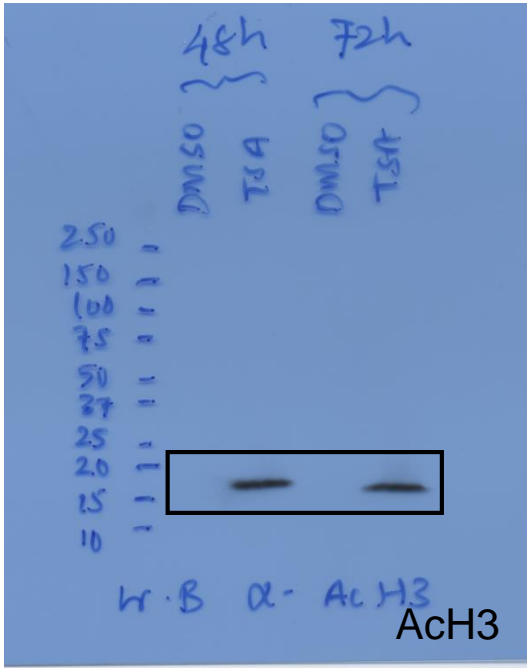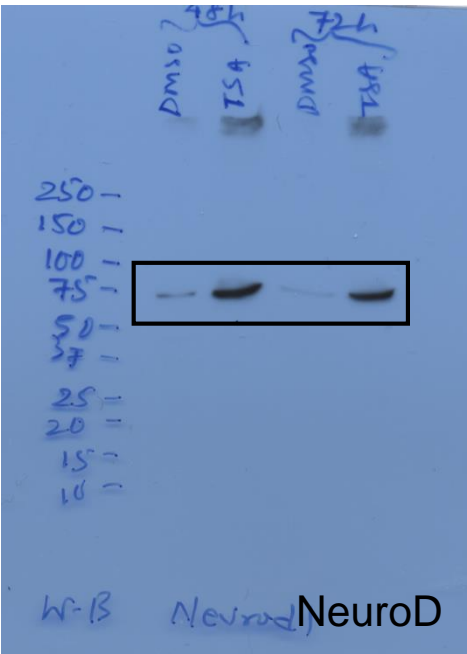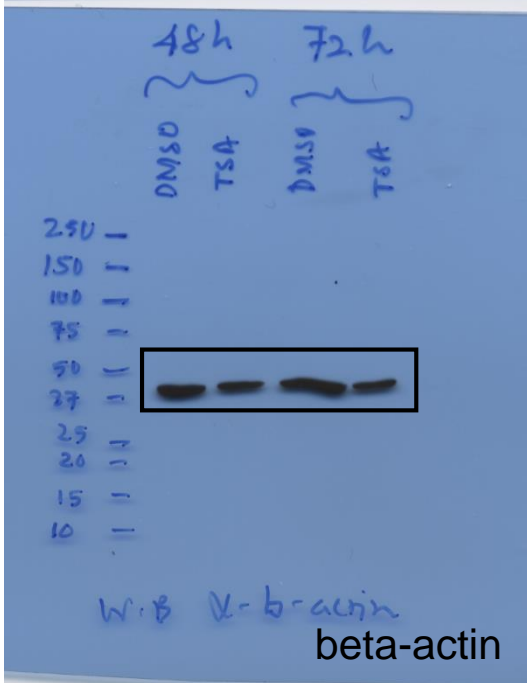

Supplement: S1 File — (PDF) [file pone.0191689.s005.pdf]
